# scnRCA: A Novel Method to Detect Consistent Patterns of Translational Selection in Mutationally-Biased Genomes

**DOI:** 10.1371/journal.pone.0076177

**Published:** 2013-10-07

**Authors:** Patrick K. O'Neill, Mindy Or, Ivan Erill

**Affiliations:** Department of Biological Sciences, University of Maryland Baltimore County (UMBC), Baltimore, Maryland, United States of America; Beijing Institute of Genomics, Chinese Academy of Sciences, China

## Abstract

Codon usage bias (CUB) results from the complex interplay between translational selection and mutational biases. Current methods for CUB analysis apply heuristics to integrate both components, limiting the depth and scope of CUB analysis as a technique to probe into the evolution and optimization of protein-coding genes. Here we introduce a self-consistent CUB index (scnRCA) that incorporates implicit correction for mutational biases, facilitating exploration of the translational selection component of CUB. We validate this technique using gene expression data and we apply it to a detailed analysis of CUB in the Pseudomonadales. Our results illustrate how the selective enrichment of specific codons among highly expressed genes is preserved in the context of genome-wide shifts in codon frequencies, and how the balance between mutational and translational biases leads to varying definitions of codon optimality. We extend this analysis to other moderate and fast growing bacteria and we provide unified support for the hypothesis that C- and A-ending codons of two-box amino acids, and the U-ending codons of four-box amino acids, are systematically enriched among highly expressed genes across bacteria. The use of an unbiased estimator of CUB allows us to report for the first time that the signature of translational selection is strongly conserved in the Pseudomonadales in spite of drastic changes in genome composition, and extends well beyond the core set of highly optimized genes in each genome. We generalize these results to other moderate and fast growing bacteria, hinting at selection for a universal pattern of gene expression that is conserved and detectable in conserved patterns of codon usage bias.

## Introduction

Protein-coding genes exhibit distinct patterns of codon usage, known as Codon Usage Bias (CUB). These patterns can be distinguished at three main levels: inter-genomic, intra-genomic and intra-genic [1], [2]. Among bacterial species, many causes are thought to influence CUB. Differences in genomic %GC content are the most obvious explanation for divergent patterns of codon usage [3], but protein-coding sequences can be subject to further mutational biases, like GC-skew [2], [4], and to genomic architectural constraints [5]. Codon usage bias is known to correlate with tRNA abundance [6], [7] and gene expression levels [8], [9], suggesting that translational selection also plays a substantial role in shaping the genomic codon usage bias [10]. It has been shown that similar principles apply to codon pairs and successive synonymous codon pairs [11], [12]. Translational selection on the CUB is believed to originate from the limited availability of ribosomes and tRNAs during fast-growth (the *throughput hypothesis*) [1], [13], [14] and/or the cost associated with missense and nonsense errors during protein translation (the *accuracy hypothesis*) [15]–[20].

Codon usage bias is typically measured using indices, which assign a single score to each protein-coding sequence under analysis [21]. Many CUB indices rely on a measurement of the deviation of a gene's codon usage from a putatively optimal codon profile. Among these, the Codon Adaptation Index (CAI) has gained the most widespread acceptance and has become a *de facto* standard for analysis of codon bias and prediction of gene expression [9], [22]–[25]. Using the observed codon frequencies in a reference set, CAI defines the weight (*w(xyz)*) of codon *xyz* (coding for amino acid *aa*) as the ratio of the observed frequency of codon *xyz* (*f_xyz_*) to that of the most frequent of the synonymous codons (*f_ijk_*) for amino acid *aa*. For any given gene sequence of length *L* (in codons), the CAI score of the sequence is defined as the geometric mean of the weights of its codons [22]:
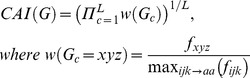
(1)


In order to predict gene expression, CAI presumes that a reference set of highly expressed genes is known [22], [26], but this is most often not the case, especially for novel species or in large-scale comparative genomics studies. This can be partially circumvented by detecting orthologs of genes known to be highly expressed in reference organisms, such as those encoding ribosomal proteins in *E. coli*, in order to generate the reference set in the organism of interest [27]. However, this method assumes that the nature of translational selection is conserved across species. Furthermore, it implicitly assumes that translational selection is the main contributor to CUB in the organism of interest and that CUB is restricted mostly to a subset of proteins. These assumptions may turn out to be false, potentially leading to flawed inferences [15], [26], [28]–[30].

A reference set can also be derived through an iterative algorithm capable of identifying a small set of genes that defines a dominating and self-consistent CUB within a given genome [31]. The resulting self-consistent CAI (scCAI) does not presume the existence of translational bias and can therefore be used to explore the nature of the dominating codon usage bias in different species [32]. However, this means that scCAI results must be analyzed with additional methods in order to determine whether the detected bias is due to translational selection or confounding factors, like %GC content [31]–[33]. Several methods have been proposed to facilitate the convergence of scCAI-like algorithms onto the translational bias [25], [33], [34], but these heuristics still make use of annotations for highly expressed genes to assist convergence. Hence they presume both the existence and the conservation of some form of translational bias, limiting their applicability to groups of organisms on which the cause and nature of translational bias are known to be conserved.

Recently we introduced a novel codon bias index, the Relative Codon Adaptation index (RCA) capable of addressing compositional biases directly in its formulation [35]:
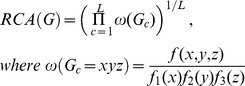
(2)


As in CAI, the RCA score of a coding sequence *G* is defined as the geometric mean of the weights of each codon along its length *L* (in codons). However, for each possible codon *xyz* the RCA weights (*ω(xyz)*) are defined as the ratio of the observed to expected frequencies of this codon on the reference set. The normalization of the *ω(xyz)* weights in RCA was shown to improve prediction of gene expression using a reference set of highly expressed genes in the compositionally biased genome of *Mycobacterium smegmatis* [35].

A drawback of CAI when integrated into the iterative algorithmic framework of scCAI is its propensity to converge onto CUB patterns unrelated with translational selection, such as %GC bias and %GC skew [31], [32]. The enhanced performance of RCA on genomes with biased composition suggested that integrating RCA within the iterative framework of scCAI could improve its convergence onto CUB patterns resulting from translational selection. In order to integrate RCA into the iterative paradigm, we first derived nRCA, a version of RCA that includes explicit normalization for amino acid usage:
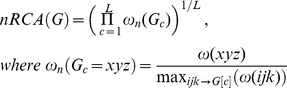
(3)


The expression for nRCA makes use of the *RCA_xyz_* weights of Equation 2 to normalize for amino acid content using the maximum synonymous codon weight, as in CAI (Equation 1), providing an index that should be resilient to both amino acid and compositional biases and can be directly integrated into the self-consistency framework of Carbone *et al*. [32], yielding the self-consistent normalized RCA, or scnRCA.

Among bacteria, differences in the pattern of codon usage are thought to be due mostly to compositional biases and, specifically, to diverging %GC-content [10]. This implies that, if present, translational selection must act in response to mutational changes in order to preserve the optimality of gene sequences through coordinated changes in the concentration of tRNA species and the codon usage patterns of gene sequences. To date, however, no study has directly assessed the effects of mutational biases on the codon repertoire and the genomic distribution of translational selection. Using scnRCA, we analyze translational bias in the Pseudomonadales, an order of taxonomically similar cold-adapted bacteria with high or moderate growth-rates and markedly dissimilar %GC content that provides a natural experiment in which to examine the adaptation of translational codon usage to genomic changes in %GC content. Our results show that scnRCA correlates well with expression data, and we use this index to validate recent reports on specific codon preferences, illuminating the tradeoffs which drive the evolution of CUB. Finally, our analysis of the Pseudomonadales reveals an unexpected degree of conservation of codon optimization across the entire genome. We extend these results to other moderate- and fast-growing bacterial in distantly related phyla, suggesting that the signature of a universal plan for gene expression among such bacteria is preserved in genome-wide codon usage profiles.

## Materials and Methods

### Genome data

Genome sequences were retrieved from the NCBI via FTP. All complete genome sequences available for the *Pseudomonas* and *Psychrobacter* families were included. Representative species of moderate and fast-growing Firmicutes, Actinobacteria and Gammaproteobacteria were selected based on previous work [32]. For species with available expression data the NCBI GenBank accession number provided with the Gene Expression Omnibus (GEO) record was used to retrieve the appropriate genome sequence.

### Gene expression data

Gene expression data for 32 bacterial species was obtained from the NCBI Gene Expression Omnibus (GEO) database [36]. Gene expression data for *E. coli* and proteome data *M. smegmatis* was obtained from the M^3D^ database [37] and Wang *et al*. [38], respectively, as described previously [35]. All gene expression data corresponds to experimental controls growing in early or mid-log phase. GEO data was downloaded in native formats, manually curated and stored in spreadsheet format for easier manipulation (Table S1).

### tRNA identification

Gene copy number for all organisms was estimated by scanning the available genome sequences using the tRNAscan-SE software with default parameters [39]. tRNA codon-anticodon pairs were assigned using the standard genetic code.

### Codon usage bias computation

Computation of codon usage bias for the scCAI and scnRCA indices was performed using the scnRCA program, a standalone Java application based on CAIJava [31]. The scnRCA program operates on GenBank genome files or FASTA formatted files containing open reading frames, and implements both the scnRCA and scCAI algorithms. The iterative algorithm begins by including all protein-coding genes within an initial reference set. CAI (w*_xyz_*) or nRCA (n*RCA_xyz_*) weights are computed and all protein-coding genes are scored using the given index. The highest scoring *D^-R^* fraction (where *D* is a pre-specified constant [2 by default] and *R* is the iteration number) is selected as the *Rth* reference set, weights are recomputed, all genes are re-scored anew and the process is iterated until the reference set reaches a predefined fraction *F* [1% by default] of the protein-coding genes in the genome. In addition, the scnRCA program allows the user to seed the algorithm with a provided reference set in FASTA format or to start the process with a randomly chosen fraction *F* of the genome. The algorithm halts when convergence is achieved (i.e. no variation in weight values between successive iterations) or a predefined maximum number of iterations (*I*) is reached. The scnRCA scores for all genes in the last iteration are output to a file. The scnRCA software and documentation are available for download at: https://github.com/erilllab/scnRCA. Computation of codon usage bias for the MILC, Ran & Higgs' δ and CDC indices was performed with a custom Python implementation of MILC and δ [40], [41] and with the C++ implementation of CDC provided in [42]. For MILC and δ, annotated “ribosomal protein” genes were used to construct the reference set in each genome. The tAI index values were calculated using a Python implementation of tAI [23]. tRNA copy numbers inferred by tRNAscan-SE were used to construct the table of *W_i_* values in tAI. The code for the tAI, MILC and δ Python implementations is available for download at: https://github.com/erilllab/oneill_et_al_2013.

### Ortholog identification

Orthologous genes were detected by computing reciprocal best BLAST hits for each pair of species examined in the study [43], [44], using BLASTP with default parameters and an E-value of 10^−10^. The analysis of sets of related species requires the identification of orthologs shared by all the species in the set. We identify such orthologs by locating *k*-cliques in a graph whose vertices are genes and whose edges denote reciprocal best BLAST hits, where *k* is the number of species. Cliques were identified using the NetworkX package in Python (http://networkx.github.com/). Routines for automating the reciprocal BLAST procedure can be found at: https://github.com/erilllab/RBLAST.

### Correlation with expression data

For each species under analysis, scnRCA/scCAI was run until convergence on the reference genome sequence and all sequences for protein-coding genes tagged as “ribosomal protein” were pooled to create the MILC and δ reference set. The scnRCA/scCAI/MILC/δ/CDC index values for each protein-coding gene in the genome were matched by NCBI locus tags to all protein-coding genes for which it expression data was available. When multiple instances of the same gene were present in the array, the same index value was assigned multiple times. For each expression dataset, Spearman rank correlation coefficients were determined independently for all available series, and the mean and standard error of the correlation coefficients were computed (Table S2).

### Evaluation of reference set term enrichment

A multi-genome analysis of term enrichment in the reference set isolated by scnRCA was performed by comparing term frequency in the reference set and the genome. The list of possible genomic terms was defined as the concatenation of the GenBank annotations [*/product* field] for every protein-coding gene in each genome, and the reference set terms as the restriction of the previous list to protein-coding genes that appear in the species' reference sets. We computed the frequencies of each word in both reference sets and genomes, defining the enrichment ratio as the ratio of the former to the latter. Terms that appeared fewer than 10 times in the entire set of genomes were omitted. The cutoff of 10 counts prevents domination of the enrichment profile by rare words in the reference sets which appear only once in the genomes; such words trivially attain the maximum possible enrichment ratio. (https://github.com/erilllab/refset_enrichment).

### Evaluation of codon preferences

Codon preference was measured as the ratio between the frequency of codons among reference set and genome protein-coding genes. For individual codons and amino acids, the average codon frequency in the reference set and in the genome was computed on a given group of species and normalized per amino acid. For N-ending codons, the occurrences of any N-ending codon for one of the two- or four-box amino acids were used to compute the relative frequency of each ending. To assess the significance of enrichment/depletion of each possible ending, reference set and genome frequency for each species were considered as paired observations for a Wilcoxon signed-rank test. Significance tests were conducted with the statistical computing package SciPy (http://www.scipy.org/), using the Benjamini-Hochberg procedure [45] to control the false discovery rate at *q* = 5%. Two- and four-box amino acid groups were analyzed separately.

### Analysis of Spearman rank correlations

Preservation of codon usage bias across multiple species was assessed by computing pair-wise Spearman rank correlations between scnRCA values over all conserved ortholog pairs. The significance of the observed correlations was evaluated by computing the two-sided p-value given the sample size for each pair of species using with the statistical computing package SciPy (http://www.scipy.org/).

Analysis of genome-wide translational selection was performed by computing the Spearman correlation between scnRCA values of orthologs or between scnRCA values and expression levels within a single species over a sliding window of 50% of the data after sorting by scnRCA value. For each dataset, a randomized control of equivalent sample size was generated from the bivariate uniform distribution and used to sample the distribution of the Spearman *ρ* statistic under the null hypothesis that the data are uncorrelated. The experimental and control distributions of the Spearman *ρ* statistic were compared by binning observations every five percentiles and computing Wilcoxon ranked-sum tests of each set of experimental observations against their respective controls.

### Code availability

All Python and R scripts used in this work are available for download at: https://github.com/erilllab/oneill_et_al_2013 under the GPL3 license.

## Results and Discussion

### scnRCA enhances the isolation of translational bias in mutationally biased genomes

To validate the hypothesis that the correction for genomic biases in scnRCA should enhance its ability to identify the effects of translational selection, we conducted a comprehensive benchmarking of scnRCA using available microarray expression data for moderate- and fast-growing bacterial species with and without compositional biases (Table S3). The efficiency of the scnRCA algorithm at isolating the translational bias reference set was assessed by computing the correlation of scnRCA scores with gene expression values, as well as previously introduced indices that measure how highly the index scores ribosomal proteins, assess its correlation with %GC content and evaluate the strength of the convergence to the reference set [31]–[33]. In all cases, the performance of the scnRCA algorithm was compared directly with the original scCAI implementation [31], with the MILC and δ indices, which use annotated ribosomal proteins to define the reference set [40], [41], and with the CDC index, which computes a direct estimate of deviation from the genomic average [42].

**Figure 1:**
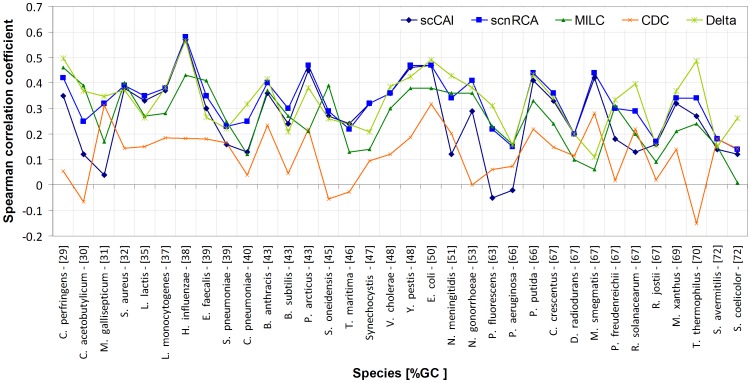
Benchmarking of codon usage bias indices with expression data. Spearman correlation of scCAI, scRCA, MILC, δ and CDC indices with expression data for different bacterial species as a function of the global %GC content of each species genome. Supporting data for this figure (number of replicates for expression values, number of annotated ribosomal proteins, etc.) is provided in Table S2.

The comparison between scCAI and scnRCA shown in Figure 1 reveals that the application of nRCA to the iterative algorithm framework systematically de-correlates the resulting index from gene %GC3 content (by 22% on average). This indicates that the content normalization inherent to scnRCA allows the iterative method to disregard compositional biases, increasing its ability to converge onto the underlying translational bias. As a result, scnRCA provides a considerable increase in correlation with expression data for genomes that exhibit compositional biases. The decrease in correlation between CUB index and genome %GC bias introduced by scnRCA can be quite dramatic, as in the case of *Mycoplasma gallisepticum* (68% decrease) or *Pseudomonas fluorescens* (63% decrease), essentially reversing the inability of scCAI to identify the translational reference set and correlate with expression data in these organisms (Figure 1). Furthermore, scnRCA can also compensate for other types of mutational bias known to hinder convergence of scnCAI, such as %GC-skew, as demonstrated by its correlation with expression data in the *Neisseriaceae*. In most cases, the improved correlation with expression data is associated with higher ribosomal scores (Table S2), which can be used as proxy for translational bias [31], [32]. Nonetheless, the results obtained for several species, such as *Bacillus subtilis* or *Chlamydophila pneumoniae*, illustrate that the de-correlation from %GC3 content is sufficient to improve correlation with expression data (Table S2). This can be true even in the absence of improved ribosomal scores, a fact that is corroborated by the comparison of scnRCA with the MILC and δ indices. These indices operate on a pre-defined reference set generated through orthology or annotation analysis of ribosomal proteins. The results of scnRCA are comparable to those of MILC and δ, further validating the notion that scnRCA is able to converge on the underlying translational selection signal in spite of substantial genomic biases. Adopting the self-consistency framework allows scnRCA to focus on the translational selection component of CUB, providing improved correlation with expression data in comparison to CDC, which is based on a statistic conceptually similar to scnRCA but is not designed to isolate translational selection (Figure 1). Even though scnRCA is not prejudiced by a predefined reference set, the results presented here indicate that its implicit correction for content biases allows it to isolate translational bias in the genomes of moderate and fast growing bacterial species without incurring the risks of directly transporting the reference set through orthology or annotation of ribosomal proteins [30]. These findings recommend scnRCA as an unbiased method for studying translational codon bias across species.

### *Pseudomonas* and *Psychrobacter* reveal consistent patterns of reference set enrichment

The genomes of *Pseudomonas* and *Psychrobacter* species present markedly divergent %GC content profiles (62.7±2.4 and 42.67±1.2, respectively) that have resulted in distinct patterns of codon usage. We exploited the improved performance of scnRCA on compositionally biased genomes to analyze the 12 available complete genome sequences of *Pseudomonas* and *Psychrobacter* species and their specific patterns of codon usage. Based on the consistent correlation of scnRCA with expression data (Figure 1), we used the self-consistent reference set identified by scnRCA as a proxy to define the set of genes under maximal translational selection (or effectome) within each organism [46]. An analysis of enrichment in gene annotation terms (Figure S1) reveals that the genetic makeup of the derived reference sets is in accordance with previous reports [27], [32], [46], [47]. As expected, ribosomal proteins and translational machinery such as elongation factors make up a significant fraction of the reference set (36-fold average enrichment). This is complemented by heat and cold shock genes, oxidative stress enzymes (e.g. superoxide dismutase) and major metabolic enzymes (e.g. succinyl-CoA synthetase). To assess the generality of these results, we analyzed the composition of the reference set inferred by scnRCA among moderate- and fast-growing species of two distant phyla with similar %GC-biases (the Actinobacteria and the Firmicutes), as well as representatives of other Gammaproteobacteria families (Table S3). In agreement with previous reports [46]–[48], none of the components identified above, including cold-shock genes, is enriched exclusively in the Pseudomonadales, suggesting that the functional composition of the effectome is largely conserved among moderate- and fast-growing bacteria.

It seems well established that, in bacteria, %GC content is one of the main drivers of codon usage bias [3], [49], but its specific interplay with translational bias is not fully understood. Codon usage bias has long been known to correlate with the concentration of tRNA species in fast-growing organisms [6], [50] and this has led some authors to propose that tRNA availability is mainly responsible for the translational component of codon usage bias [51]. Comparative genomics analyses have also revealed that the set of most abundant tRNAs remains fairly constant among bacteria, in spite of significant changes to genome %GC content [52]. Abundant tRNAs favor U and avoid A in the first anticodon position, seemingly opting for anticodons capable of reading the maximum number of synonymous codons [52]. This suggests a framework in which genomic %GC content modulates a basic organizational plan laid out by tRNA preferences [49], [52], [53].

**Figure 2:**
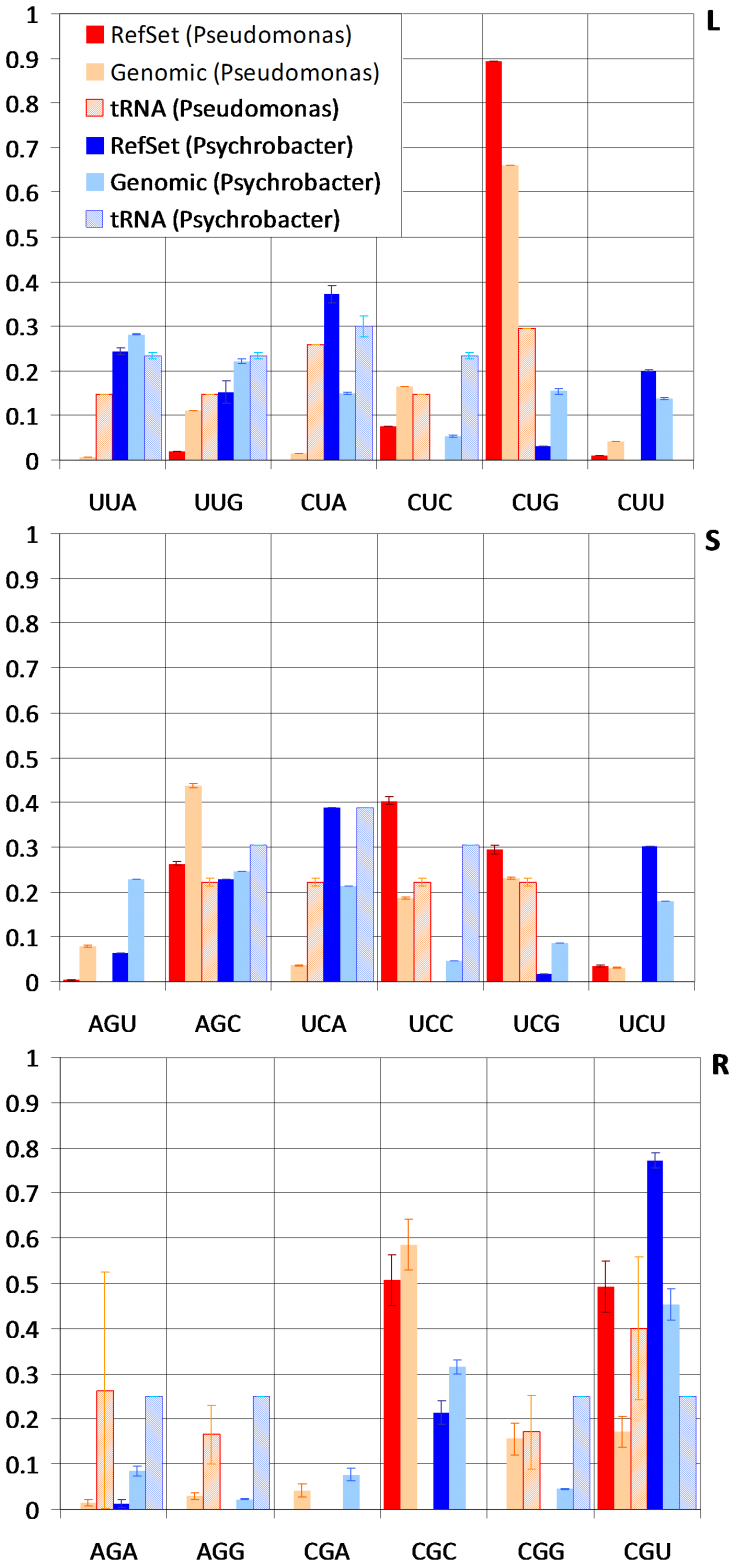
Codon and tRNA frequency distribution among six-box amino acids. Average amino acid-normalized frequencies of codons in the reference set, of codons in all protein-coding genes and of gene copy number for their cognate tRNAs. For each codon, the three leftmost series correspond to values for *Pseudomonas* species and the three rightmost to average values for *Psychrobacter* species. The amino acid is displayed on the top right. Vertical bars indicate the standard error of the mean.

Our analysis of the Pseudomonadales using an unbiased CUB index provides a unique window into the interplay between translational selection, tRNA gene copy number and compositional bias. The influence of genomic %GC content on codon usage is clearly visible when analyzing the %GC content of the derived reference sets. In both groups, the %GC content of reference sets (59.4±0.6% for *Pseudomonas* and 44.5±0.09% for *Psychrobacter*) is well in line with the genomic average (62.7±2.4 and 42.67±1.2, respectively). This has led to distinct patterns of codon usage, resulting in a moderate correlation in reference set codon frequencies (ρ*_s_* = 0.50±0.01) between the two genera. A more detailed analysis, however, reveals both significant differences and similarities in the adaptive strategies of both groups. The most substantial differences are located in amino acids encoded by 6 synonymous codons (R, S and L; Figure 2). The *Pseudomonas*, for instance, show a preference for the two-box codon AGC to code for S in the genome, and for the four-box codon UCC to encode this amino acid in the reference set, whereas the *Psychrobacter* rely on UCA and UCU for the reference set and appear to use AGU, AGC, UCA and UCU similarly for the genome. More dramatically, the *Pseudomonas* exploit the four-box CUG codon almost exclusively to code for L, in stark contrast to the *Psychrobacter*. This correlates well with the presence of tRNA(CAG) genes in the *Pseudomonas*, which are absent in all *Psychrobacter* species. However, we find that the overall correlation between tRNA copy number and the rate at which codons are preferentially used in the reference set is not very strong (ρ*_s_* = 0.29±0.03 and ρ*_s_* = 0.32±0.02 for *Pseudomonas* and *Psychrobacter*, respectively). In accordance with previous results [52], tRNA copy number remains fairly constant, with only a weak pattern of tRNA gene loss and gain trailing the dominating %GC bias. As a consequence, we obtained weaker and less consistent correlations (Δρ*_s_* = −0.17±0.03) with gene expression on both groups when applying the tAI index, based on tRNA abundance [23], than with scnRCA.

**Figure 3:**
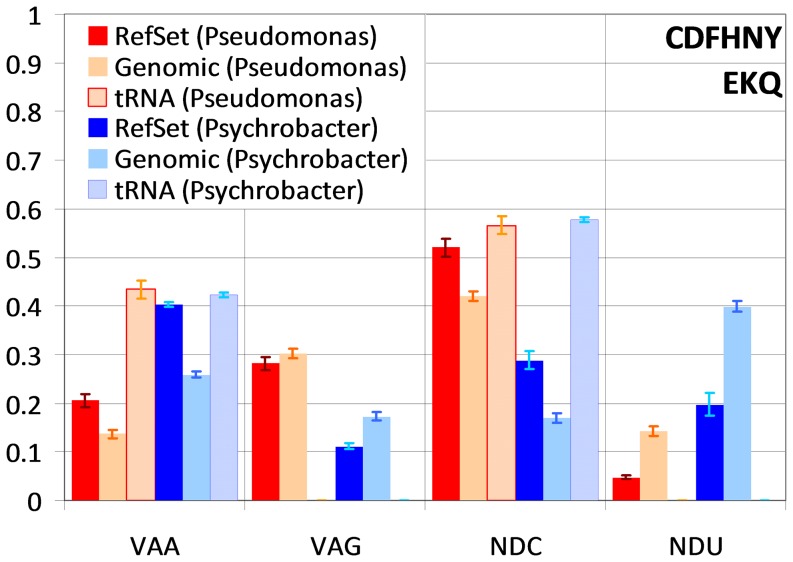
Average codon and tRNA frequency distribution for two-box amino acids. Average two-box codon-normalized frequencies of different ending codons in the reference set and in all protein-coding genes, and of gene copy number for the different ending cognate tRNAs. For each codon, the three leftmost series correspond to values for *Pseudomonas* species and the three rightmost to average values for *Psychrobacter* species. Different codon endings are denoted by the corresponding IUB representation. The respective amino acids are displayed on the bottom right. Vertical bars indicate the standard error of the mean.

**Figure 4:**
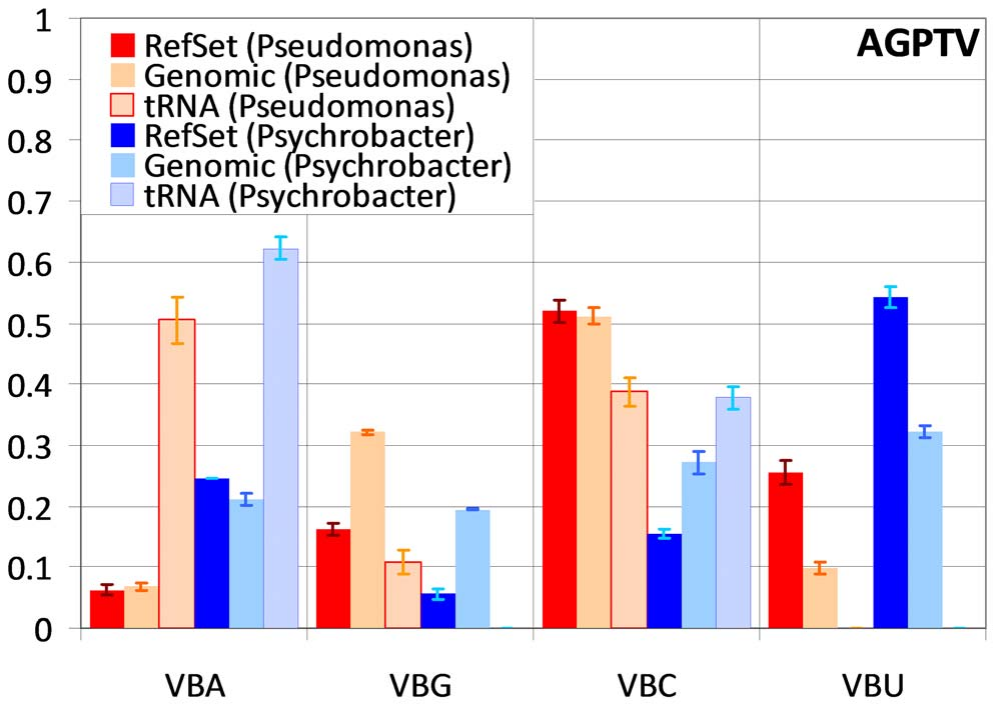
Average codon and tRNA frequency distribution for four-box amino acids. Average four-box codon-normalized frequencies of different ending codons in the reference set and in all protein-coding genes, and of gene copy number for the different ending cognate tRNAs. For each codon, the three leftmost series correspond to values for *Pseudomonas* species and the three rightmost to average values for *Psychrobacter* species. Different codon endings are denoted by the corresponding IUB representation. The respective amino acids are displayed on the bottom right. Vertical bars indicate the standard error of the mean.

In order to match the %GC content of the genome, reference sets in *Pseudomonas* and *Psychrobacter* must necessarily use different sets of preferred codons. For instance, C-ending codons show a consistently higher frequency in both the genome and reference set of *Pseudomonas*, whereas U-ending codons are much more frequent in *Psychrobacter*. Despite these differences, we observe consistent trends of reference set codon enrichment (defined as the ratio of frequencies for each codon between reference set and genome) in both groups. Among two-box amino acids (C, D, F, H, N, Y, Q, E and K; Figure 3; Figure S2), C- and A-ending codons are enriched in the reference set (Wilcoxon signed-rank test p<0.01 in both cases). The same pattern of C-ending enrichment is observed for the three-box amino acid isoleucine (I; Figure S3). This is in agreement with previous results and with the presence in all these genomes of genes for cognate tRNAs of C- and A-ending codons, and the corresponding absence of genes for cognate tRNAs of U- and G-ending codons [13], [52], [54]–[57]. In fact, there is a substantial correlation between reference set enrichment and cognate tRNA copy number for two-box amino acid codons (ρ*_s_* = 0.69±0.03). This trend is reversed in four-box amino acids (A, G, P, T and V; Figure 4; Figure S4), where U-ending codons are preferentially used in the reference set (Wilcoxon signed-rank test p<0.01) [58] [41]. In comparison to two-box amino acids, the enrichment of U-ending codons might appear counterintuitive because there are no cognate tRNAs for these codons, which are recognized by modified tRNAs, in either group of species. Accordingly, correlation between reference set enrichment and cognate tRNA copy number for four-box amino acid codons is weak and negative (ρ*_s_* = −0.13±0.03).

Four-box amino acids in the Pseudomonadales hence provide a clear picture of the complex interplay between compositional bias and translational selection and its projection as a difference in magnitude between codon frequency and enrichment. When coding for threonine (T), for instance, the *Pseudomonas* show two-fold enrichment for codon ACU, yet ACC remains the most frequent codon (82%) in highly expressed genes, as one would expect given the high genomic %GC content of these organisms (Figure S4). Conversely, the *Psychrobacter* display a similar two-fold enrichment for ACU, but in this case the selective enrichment leads to ACU being the most frequent threonine codon (62%) among highly expressed genes. This disparity in magnitudes and trends helps to explain why some of these patterns were not detected earlier and illuminates the history of conflicting arguments regarding the strength of mutational and translational biases. Depending on how “optimal codons” are defined and what method is used to infer codon usage bias, one may conclude that codon usage bias is predominantly dictated by %GC content [49], [53] or translational selection [13], [58]. The results of codon enrichment analysis can be extended to the full group of Firmicutes, Actinobacteria and Gammaproteobacteria without loss of generality. In this larger set, we observe also a preference for C- and A-ending codons (p<0.01 in both cases) and positive correlation with tRNA copy number (ρ*_s_* = 0.71±0.02) in two-box amino acids, as well as a preference for U-ending codons (p<0.01) and negative correlation with tRNA copy number (ρ*_s_* = −0.06±0.03) in four-box amino acids. The use of scnRCA hence enables us for the first time to simultaneously detect in a single analysis all previously reported two-box and four-box patterns of enrichment across a broad range of phyla and mutational biases, corroborating the insights on codon optimality drawn from the Pseudomonadales and further validating the use of scnRCA as a tool to probe into the effects of translational selection on genomic sequences.

### The genome-wide distribution of scnRCA values shows significant conservation across bacteria

**Figure 5:**
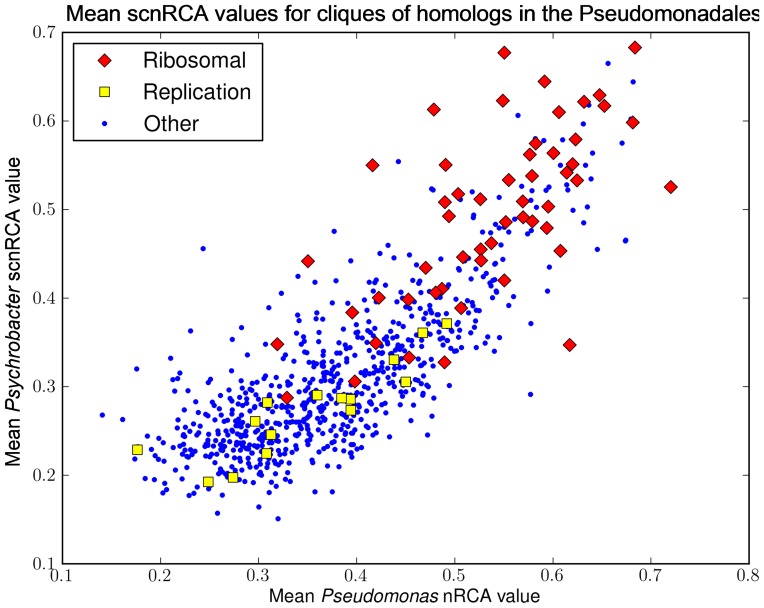
Correlation in scnRCA scores between the *Pseudomonas* and the *Psychrobacter*. Plot of average scnRCA scores for each of the 791 identified conserved homologs between *Pseudomonas* and *Psychrobacter* species. For each axis, scnRCA values correspond to the average among all species with available complete genome sequences in the represented genus. Genes corresponding to ribosomal proteins and replication-associated proteins (e.g. DNA polymerase), as determined by annotation tags, are identified with different markers. Ribosomal proteins were defined as those having the term “ribosomal protein” in its GenBank annotation. Replication-associated proteins were defined as those having any of the following terms in their annotation: “chromosome replication”, “chromosome segregation”, “DNA gyrase”, “DNA polymerase” and “DNA topoisomerase”.

The substantial difference in genomic %GC content for *Pseudomonas* and *Psychrobacter* species imposes different constraints on codon usage bias within and among protein coding genes. Having analyzed the effect of this mutational bias on specific codon preferences, we used the computed scnRCA values to evaluate the impact of %GC content on the genome-wide organization of translational bias. Using reciprocal best BLAST hits to define homologs among all *Pseudomonas* and *Psychrobacter* species, we analyzed the genome-wide distribution of scnRCA scores among 791 conserved homologs. The results shown in Figure 5 reveal a remarkable correlation between the scnRCA values of conserved homologs of *Pseudomonas* and *Psychrobacter*, with a Spearman correlation coefficient of ρ*_s_* = 0.81. In light of the divergent %GC of the two genera, this correlation cannot merely be due to sequence-level conservation. Furthermore, analysis of gene expression data for the four Pseudomonadales species for which datasets are available shows that pair-wise species correlation coefficients for expression data are also high, even if lower than those observed for scnRCA (Table S4). This difference is most likely due to the noisy nature of array data and, in particular, to low correlation values between *P. aeruginosa* and other Pseudomonadales [23]. These results indicate that scnRCA values capture a relevant component of gene expression that is preserved among genomes, providing a more resilient indicator of translational selection.

**Figure 6:**
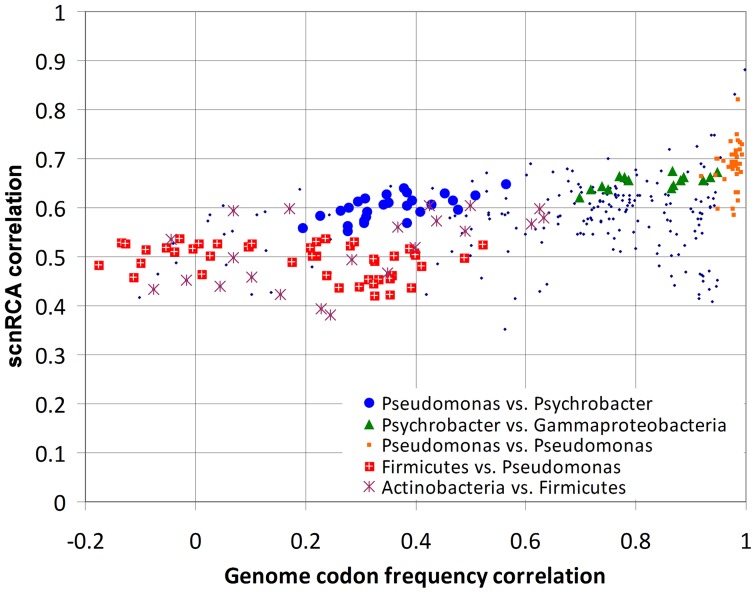
Species pair-wise correlation in scnRCA values vs. pair-wise correlation in genome codon frequency. Plot of pair-wise Spearman correlations coefficients among all species for both scnRCA values in orthologous genes (Y-axis) and genome codon frequencies (X-axis). Correlation of scnRCA values for each pair of species was computed on all available orthologs (i.e. as in Figure 5). For each codon and genome, the genome codon frequency is defined as the codon frequency over all protein-coding genes in each genome. Correlation in genome codon frequencies was computed on the 61-component genome codon frequency vector of each species. Several group-specific pairings (e.g. Firmicutes vs. *Pseudomonas*) are highlighted using different markers.

**Figure 7:**
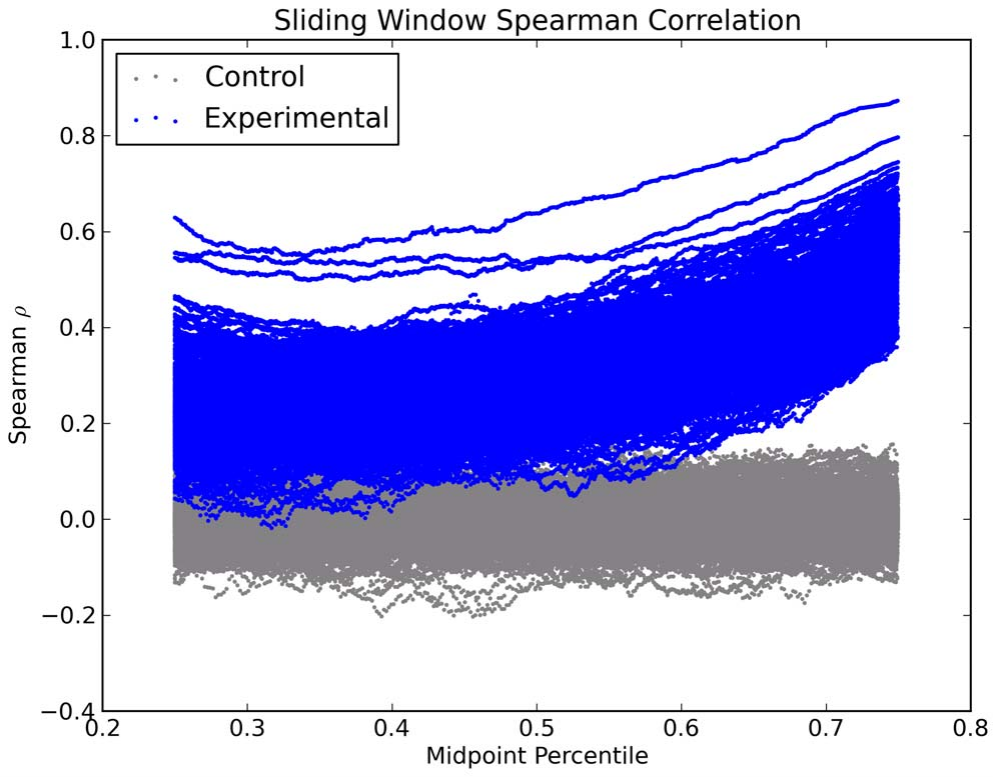
Sliding-window analysis of inter-species pair-wise correlation. Plot of pair-wise Spearman correlations coefficients (blue) among all species for scnRCA values (Y-axis) in orthologous genes as a function of the position of the center of a sliding window (X-axis) spanning half the total number of pair-wise conserved homologs, sorted by scnRCA value. The leftmost point on the X-axis corresponds to the window encompassing the lowest half of the scnRCA values among the orthologs between any two given species. The rightmost point on the X-axis corresponds to the window encompassing the highest half of the scnRCA values among the orthologs between any two given species. Correlation of scnRCA values for each pair of species was computed on all available orthologs between both species. Window positions have been normalized to the total number of conserved homologs in each pair of species to allow consistent overlaying. The p-values associated with each Spearman correlation are reported in Table S5. For each set of pair-wise homologs a randomized control of equal sample size is also shown (grey). The difference between the observed and control distributions of the Spearman *ρ* statistic are statistically significant across the whole range of scnRCA values. The results of Wilcoxon signed-rank tests against the paired randomized controls are reported in Table S7.

To gauge the extent to which scnRCA values are conserved among more distantly related species, we analyzed the correlation of scnRCA values among the previously defined set of moderate- and fast-growing species (Table S3). Figure 6 plots the pair-wise species Spearman correlation coefficients for scnRCA values as a function of the correlation in genome codon frequencies for each pair. As expected, organisms belonging to the same phylogenetic group present the strongest correlations, both in terms of scnRCA values and genome codon frequencies. For instance, the *Pseudomonas* present an average ρ*_s_* of 0.68±0.006 for scnRCA values and of 0.98±0.002 for genome codon frequencies. More generally, the plot also shows evidence of increasing correlation in scnRCA values as a result of closer genome codon frequencies (ρ*_s_* = 0.56, Pearson *r* = 0.54), but the slope of the linear regression is small (*β* = 0.14) and induced mostly by within-group correlations, such as the *Pseudomonas*, discussed above. This moderate dependency of scnRCA correlation on codon frequencies is illustrated by the relationship between the *Psychrobacter* and the Gammaproteobacteria, which generates two distinct clusters in Figure 6. *Psychrobacter* species present significantly better correlation in terms of codon frequency with other low %GC Gammaproteobacteria, like *Haemophilus influenzae* or *Vibrio cholerae*, but this translates only into a marginal increase in scnRCA correlation (from an average ρ*_s_* of 0.60±0.005 to 0.66±0.004). Overall, the plot shows a remarkable stability in the pair-wise correlation of scnRCA values across genomes (ρ*_s_* = 0.56±0.004) even among distantly related species with negatively correlated codon usage profiles and genome %GC contents, such as the *Pseudomonas* and the low %GC Firmicutes. To test whether this effect was due solely to correlation in scnRCA values among highly expressed genes, we performed a sliding-window analysis of inter-species pair-wise correlations, which reveals the contribution to the observed Spearman correlation (Figure 6) from different intervals of scnRCA values. The sliding-window analysis (Figure 7) reveals that the scnRCA correlation signal is present and statistically significant throughout the whole range of scnRCA values (Table S5; Table S7; Figure S5). Correlation coefficients peak for high scnRCA ranges, but maintain on average significantly large values for the low scnRCA ranges, suggesting that the whole genome is under selection for CUB optimization. This result is supported by an equivalent sliding-window analysis of the correlation of scnRCA with expression values for the species analyzed here, which also reveals lower but consistent and statistically significant correlation of scnRCA values with expression across the whole range of scnRCA values (Figure S6; Table S6; Table S7).

To date, most genomic analyses of CUB across bacteria have focused on a subset of highly optimized genes that can be set apart from the rest of the genome by algorithmic or statistical means. For the most part, this core set of genes, or effectome, has been analyzed by means of Gene Ontology (GO), Clusters of Orthologous Genes (COGs) or gene annotation word counts (Figure S1), leading to qualitative assessments of the function and evolutionary mechanism for selection of these genes [46]–[48], [59]. Here we use scnRCA to conduct for the first time a systematic analysis of conservation of CUB patterns across multiple bacterial species. The stability of the observed correlations in scnRCA values and their presence throughout the entire range of scnRCA values provide evidence that translational selection operates consistently on the whole genome. Even though models for the evolution for CUB predict that the whole genome should be under selection for CUB, the usually weak correlation of genes with low index values with noisy expression estimates has prevented a thorough investigation into the consistency of this effect [23], [60]. The genome-wide analysis of conservation of CUB across distant genomes with divergent %GC content using an unbiased estimator of CUB provides the means to analyze directly the degree of translational selection across the genome. Our results indicate that translational selection is stronger than conventionally assumed among genes not belonging to the effectome [1], [47]. Furthermore, the presence of correlation in CUB values across the genome points to the existence of a shared organizational plan for gene expression among moderate and fast-growing bacteria. This agrees with and expands on previous results based on the compositional analysis of effectomes [46], [47]. Our results show that the stability in effectome composition reported in previous work extends spatially to encompass the whole genome, but also logically to incorporate preservation of specific optimization arrangements across genomes. With hindsight, the existence of such an organizational plan might seem like a foregone conclusion; moderate- and fast-growing bacteria obviously share a set of basic metabolic requirements and constraints related to growth and it is therefore to be expected that these should be reflected in their genetic organization. However, conservation of broad gene expression patterns need not necessarily result in highly correlated CUB values. Our results therefore hint at a fine-grained structure to the basic expression profile of moderate- and fast-growing bacteria and pave the way for further analyses of its connection with metabolic and growth processes.

## Supporting Information

Figure S1 **Word cloud of enriched annotation terms for the Pseudomonadales.** The word cloud was generated by constructing a list of terms in which the count of each word is proportional to its enrichment ratio, using the worldle.net web service (http://www.wordle.net/). Notable is the predominance of terms associated with translational machinery; the heat-, cold- and oxidative-shock responses; and central carbon metabolism.
(PDF)

Figure S2 **Codon and tRNA frequency distribution for two-box amino acids.** Average two-box amino acid-normalized frequencies for codons in the reference set and in all protein-coding genes, and of gene copy number for the different ending cognate tRNAs. For each codon, the three leftmost series correspond to values for *Pseudomonas* species and the three rightmost to average values for *Psychrobacter* species. The respective amino acids are displayed on the top right. Vertical bars indicate the standard error of the mean.
(PDF)

Figure S3 **Codon and tRNA frequency distribution for isoleucine.** Average isoleucine-normalized frequencies for codons in the reference set and in all protein-coding genes, and of gene copy number for the different ending cognate tRNAs. For each codon, the three leftmost series correspond to values for *Pseudomonas* species and the three rightmost to average values for *Psychrobacter* species. Vertical bars indicate the standard error of the mean.
(PDF)

Figure S4 **Codon and tRNA frequency distribution for four-box amino acids.** Average four-box amino acid-normalized frequencies for codons in the reference set and in all protein-coding genes, and of gene copy number for the different ending cognate tRNAs. For each codon, the three leftmost series correspond to values for *Pseudomonas* species and the three rightmost to average values for *Psychrobacter* species. The respective amino acids are displayed on the top right. Vertical bars indicate the standard error of the mean.
(PDF)

Figure S5 **Distribution of nRCA values for orthologous cliques.** Distributions of nRCA values for orthologous cliques shared between the full set of bacterial species analyzed in this work, sorted by median value.
(PDF)

Figure S6 **Sliding-window analysis of correlation between scnRCA and expression levels.** Plot of pair-wise inter-species Spearman correlations coefficients (Y-axis, blue) assessing the correlation between scnRCA values and expression levels as a function of the position of the center of a sliding window (X-axis) spanning half the total number of pair-wise conserved homologs sorted by scnRCA value. The leftmost point on the X-axis corresponds to the window encompassing the lowest half of the scnRCA values among the orthologs between any two given species. The rightmost point on the X-axis corresponds to the window encompassing the highest half of the scnRCA values among the orthologs between any two given species. Correlation of scnRCA values with expression data was assessed on all 32 species for which expression data was available (Table S1). Window positions have been normalized to the total number of conserved homologs in each species pair to allow consistent overlaying. The p-values associated with each Spearman correlation are reported in Table S6. For each set of pair-wise homologs a randomized control of equal sample size is also shown (grey). The difference between the observed and control distributions of the Spearman *ρ* statistic are statistically significant across the whole range of scnRCA values. The results of Wilcoxon signed-rank tests against the paired randomized controls are reported in Table S7.
(PDF)

Table S1 **Gene expression data for 32 bacterial species.** Gene expression data was obtained from GEO, selecting for control samples in experiments performed on exponential-phase growth, and mapped to GenBank CDS locus tags.
(CSV)

Table S2 **Index correlation benchmark with expression data.** Spearman correlation of scCAI, scRCA, MILC, CDC and Ran & Higgs' δ with expression data for different bacterial species. The average and standard error of the Spearman correlation *r_s_*, number of array samples (*S#*) and replicates (*R#)* and GEO accession number, are shown together with the ribosomal (*Rib.*), strength (*Str.*) and %GC3 content (*Cont.*) criteria for the scCAI and scnRCA methods, the number of annotated ribosomal proteins (RP#) used for the MILC method, and the genomic %GC content of each species. The ribosomal criterion (*Rib.*) is computed as the mean Z-score of scCAI/scnRCA values for ribosomal proteins against scCAI/scnRCA values for all protein-coding genes in the genome. The strength criterion (*Str.*) is computed as the mean Z-score of scCAI/scnRCA values for the isolated reference set against scCAI/scnRCA values for all protein-coding genes in the genome. The %GC3 content (*Cont.*) criterion is computed as the correlation between scCAI/scnRCA values and gene %GC3 content for all protein-coding genes in the genome (see Carbone *et al*. (2003) *Bioinformatics*,19:16, 2005-2015).
(PDF)

Table S3 **Doubling times for bacterial species.** Minimal doubling times and reference PMID numbers for the bacterial species analyzed in this work. Doubling times were extracted from the literature. Bacteria were defined as moderate-growing if their doubling times were less than 6 h, and fast-growing if their doubling times were less than 1 h.
(CSV)

Table S4 **Pair-wise correlation in expression and scnRCA values.** Expression and scnRCA pair-wise Spearman correlation coefficients among *Pseudomonas* and *Psychrobacter* species.
(CSV)

Table S5 **Statistical significance of pair-wise Spearman rank correlation coefficients for scnRCA –scnRCA correlations.** Spearman rank correlation coefficients and associated p-values for all the Spearman rank correlation coefficients used to generate Figure 7.
(CSV)

Table S6 **Statistical significance of pair-wise Spearman rank correlation coefficients for scnRCA-expression correlations.** Spearman rank correlation coefficients and associated p-values for all the Spearman rank correlation coefficients used to generate Figure S6.
(CSV)

Table S7 **Statistical significance of pair-wise correlations in expression and scnRCA values.** Distributions of Spearman rank correlation coefficients for pair-wise scnRCA-scnRCA and scnRCA-expression correlations in Figure 7 and Figure S6 (respectively) are compared to the null distribution of correlation coefficients by generating for each dataset a randomized, uncorrelated control dataset of equal size. Experimental and control correlation coefficients were binned by the midpoint of the sliding window and compared using Wilcoxon signed-rank tests. The reported *T* statistic is the minimum of the sums of the signed ranks greater or lesser than zero, as given in the SciPy implementation of the Wilcoxon test.
(CSV)
