# Supplementary figures and images for: scnRCA: A Novel Method to Detect Consistent Patterns of Translational Selection in Mutationally-Biased Genomes
Source: PLoS One. 2013 Oct 7;8(10):e76177. doi: 10.1371/journal.pone.0076177 (PMC3792112; doi:10.1371/journal.pone.0076177)

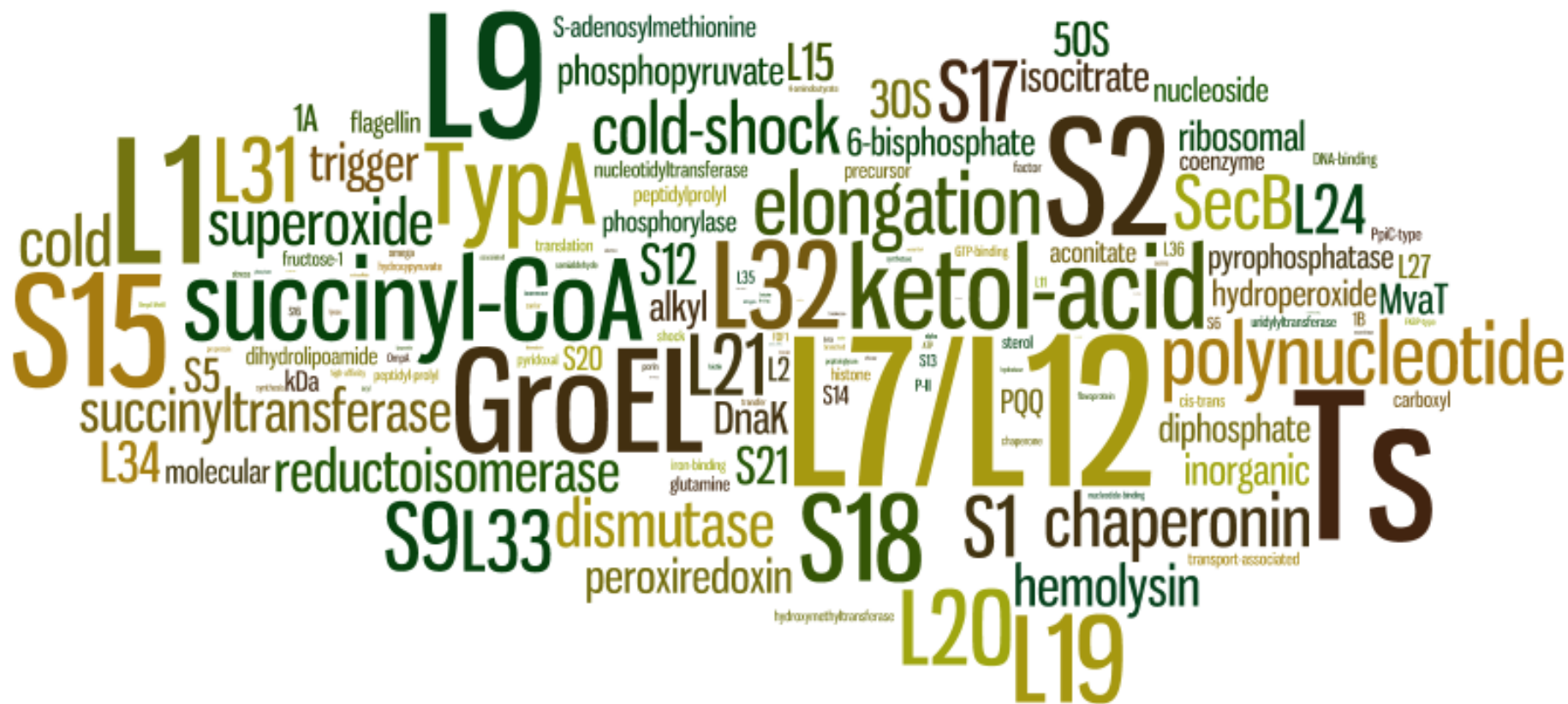

**Figure S1 – Word cloud of enriched annotation terms for the Pseudomonadales.**

Supplement: Figure S1 — Word cloud of enriched annotation terms for the Pseudomonadales. The word cloud was generated by constructing a list of terms in which the count of each word is proportional to its enrichment ratio, using the worldle.net web service (http://www.wordle.net/). Notable is the predominance of terms associated with translational machinery; the heat-, cold- and oxidative-shock responses; and central carbon metabolism. (PDF) [file pone.0076177.s001.pdf]
