# Supplementary material for: scnRCA: A Novel Method to Detect Consistent Patterns of Translational Selection in Mutationally-Biased Genomes
Source: PLoS One. 2013 Oct 7;8(10):e76177. doi: 10.1371/journal.pone.0076177 (PMC3792112; doi:10.1371/journal.pone.0076177)
Supplement: Figure S4 — Codon and tRNA frequency distribution for four-box amino acids. Average four-box amino acid-normalized frequencies for codons in the reference set and in all protein-coding genes, and of gene copy number for the different ending cognate tRNAs. For each codon, the three leftmost series correspond to values for Pseudomonas species and the three rightmost to average values for Psychrobacter species. The respective amino acids are displayed on the top right. Vertical bars indicate the standard error of the mean. (PDF) [file pone.0076177.s004.pdf]

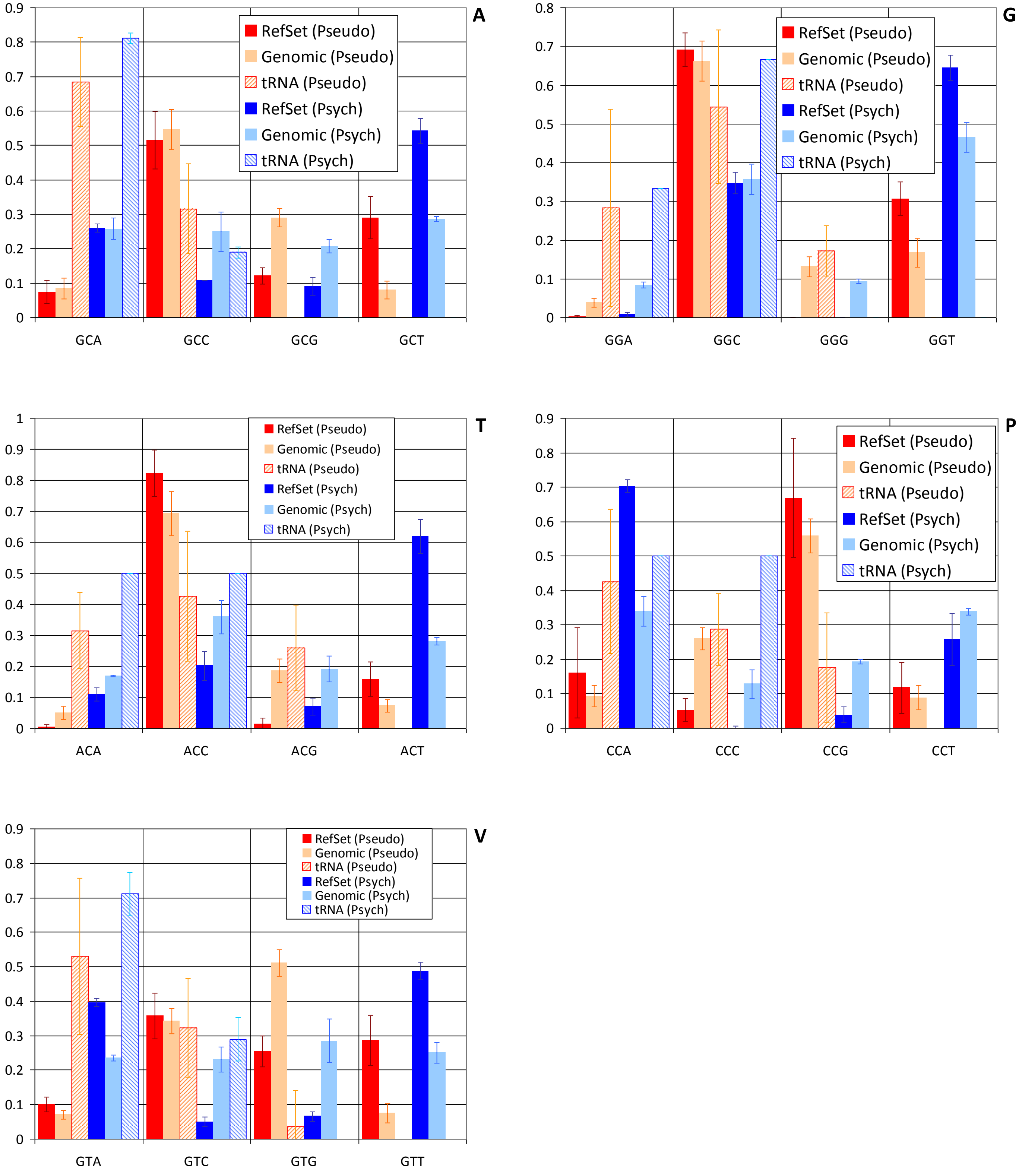

Figure S4 – Codon and tRNA frequency distribution for four-box amino acids.
Average four-box amino acid-normalized frequencies for codons in the reference set and in all protein-coding genes, and of gene copy number for the different ending cognate tRNAs. For each codon, the three leftmost series correspond to values for *Pseudomonas* species and the three rightmost to average values for *Psychrobacter* species. The respective amino acids are displayed on the top right. Vertical bars indicate the standard error of the mean.
