# Supplementary material for: scnRCA: A Novel Method to Detect Consistent Patterns of Translational Selection in Mutationally-Biased Genomes
Source: PLoS One. 2013 Oct 7;8(10):e76177. doi: 10.1371/journal.pone.0076177 (PMC3792112; doi:10.1371/journal.pone.0076177)
Supplement: Figure S5 — Distribution of nRCA values for orthologous cliques. Distributions of nRCA values for orthologous cliques shared between the full set of bacterial species analyzed in this work, sorted by median value. (PDF) [file pone.0076177.s005.pdf]

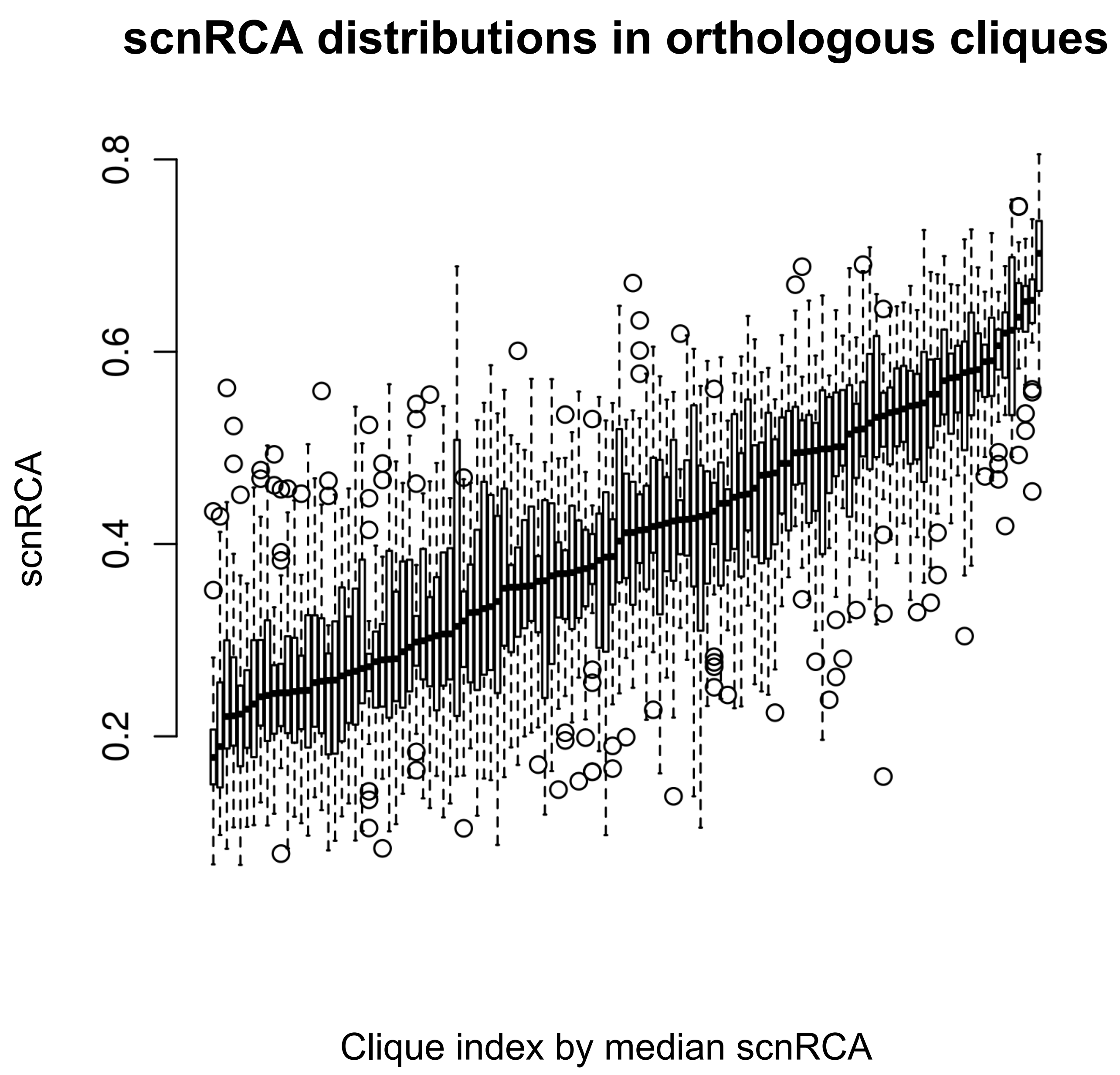

Figure S5 – Distribution of scnRCA values for orthologous cliques.
Distributions of scnRCA values for orthologous cliques shared between the full set of bacterial species analyzed in this work, sorted by median value.
