# Supplementary material for: scnRCA: A Novel Method to Detect Consistent Patterns of Translational Selection in Mutationally-Biased Genomes
Source: PLoS One. 2013 Oct 7;8(10):e76177. doi: 10.1371/journal.pone.0076177 (PMC3792112; doi:10.1371/journal.pone.0076177)
Supplement: Figure S6 — Sliding-window analysis of correlation between scnRCA and expression levels. Plot of pair-wise inter-species Spearman correlations coefficients (Y-axis, blue) assessing the correlation between scnRCA values and expression levels as a function of the position of the center of a sliding window (X-axis) spanning half the total number of pair-wise conserved homologs sorted by scnRCA value. The leftmost point on the X-axis corresponds to the window encompassing the lowest half of the scnRCA values among the orthologs between any two given species. The rightmost point on the X-axis corresponds to the window encompassing the highest half of the scnRCA values among the orthologs between any two given species. Correlation of scnRCA values with expression data was assessed on all 32 species for which expression data was available (Table S1). Window positions have been normalized to the total number of conserved homologs in each species pair to allow consistent overlaying. The p-values associated with each Spearman correlation are reported in Table S6. For each set of pair-wise homologs a randomized control of equal sample size is also shown (grey). The difference between the observed and control distributions of the Spearman ρ statistic are statistically significant across the whole range of scnRCA values. The results of Wilcoxon signed-rank tests against the paired randomized controls are reported in Table S7. (PDF) [file pone.0076177.s006.pdf]

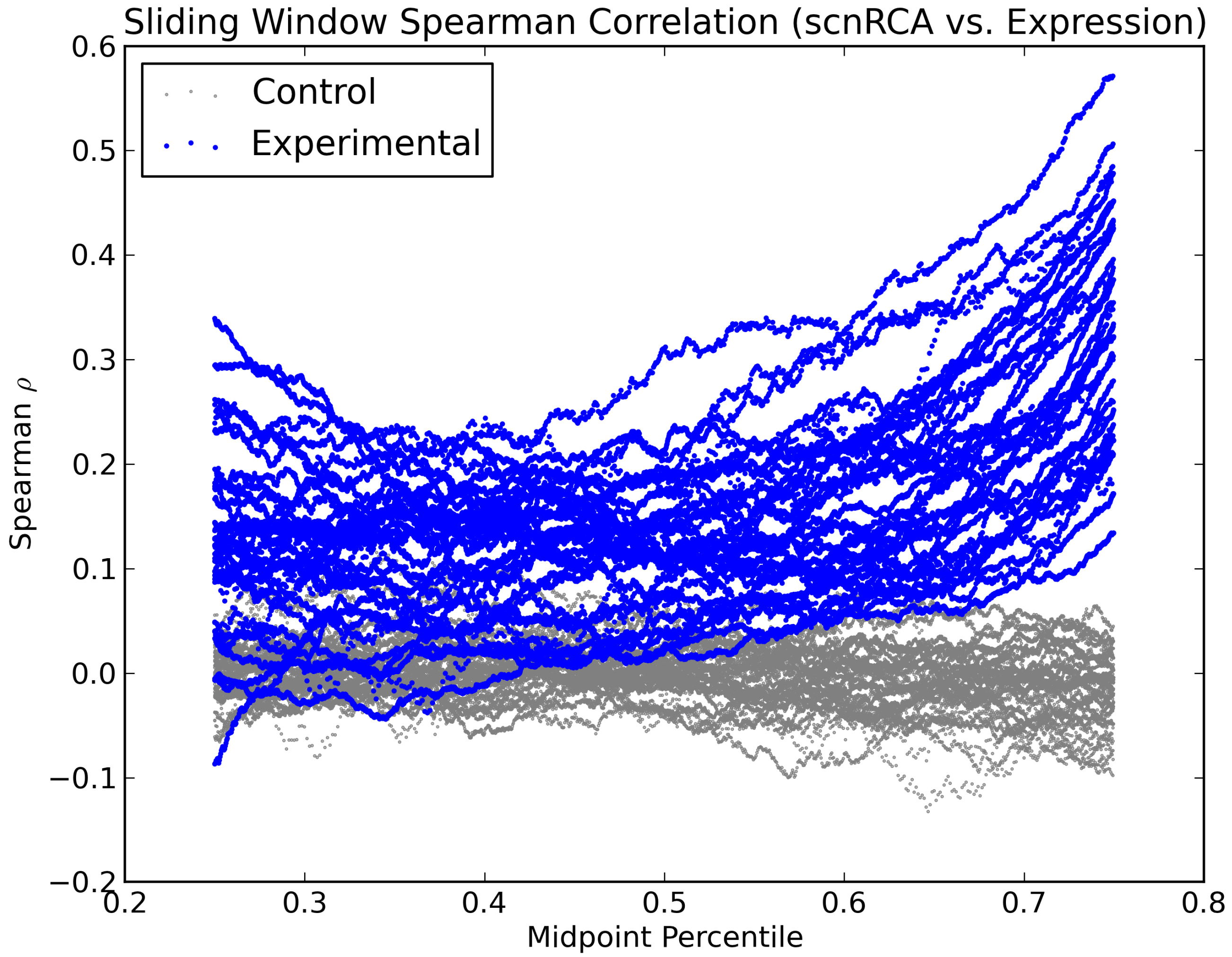

Figure S6 – Sliding-window analysis of correlation between scnRCA and expression levels.
Plot of pair-wise inter-species Spearman correlations coefficients (Y-axis, blue) assessing the correlation between scnRCA values and expression levels in orthologous genes as a function of the position of the center of a sliding window (X-axis) spanning half the total number of pair-wise conserved homologs sorted by scnRCA value. The leftmost point on the X-axis corresponds to the window encompassing the lowest half of the scnRCA values among the orthologs between any two given species. The rightmost point on the X-axis corresponds to the window encompassing the highest half of the scnRCA values among the orthologs between any two given species. Correlation of scnRCA values with expression data was assessed on all 32 species for which expression data was available (Table S1). Window positions have been normalized to the total number of conserved homologs in each species pair to allow consistent overlaying. The p-values associated with each Spearman correlation are reported in Table S6. For each set of pair-wise homologs a randomized control of equal sample size is also shown (grey). The difference between the observed and control distributions of the Spearman ρ statistic are statistically significant across the whole range of scnRCA values. Results of Wilcoxon signed-rank tests against the paired randomized controls are reported in Table S7.
