# Supplementary material for: scnRCA: A Novel Method to Detect Consistent Patterns of Translational Selection in Mutationally-Biased Genomes
Source: PLoS One. 2013 Oct 7;8(10):e76177. doi: 10.1371/journal.pone.0076177 (PMC3792112; doi:10.1371/journal.pone.0076177)
Supplement: Table S2 — Index correlation benchmark with expression data. Spearman correlation of scCAI, scRCA, MILC, CDC and Ran & Higgs' δ with expression data for different bacterial species. The average and standard error of the Spearman correlation rs, number of array samples (S#) and replicates (R#) and GEO accession number, are shown together with the ribosomal (Rib.), strength (Str.) and %GC3 content (Cont.) criteria for the scCAI and scnRCA methods, the number of annotated ribosomal proteins (RP#) used for the MILC method, and the genomic %GC content of each species. The ribosomal criterion (Rib.) is computed as the mean Z-score of scCAI/scnRCA values for ribosomal proteins against scCAI/scnRCA values for all protein-coding genes in the genome. The strength criterion (Str.) is computed as the mean Z-score of scCAI/scnRCA values for the isolated reference set against scCAI/scnRCA values for all protein-coding genes in the genome. The %GC3 content (Cont.) criterion is computed as the correlation between scCAI/scnRCA values and gene %GC3 content for all protein-coding genes in the genome (see Carbone et al. (2003) Bioinformatics,19:16, 2005-2015). (PDF) [file pone.0076177.s008.pdf]

Table S2 – Index correlation benchmark with expression data.

Spearman correlation of scCAI, scRCA, MILC, CDC and Ran & Higgs' δ with expression data for different bacterial species. The average and standard error of the Spearman correlation r_s, number of array samples (*S#*) and replicates (*R#)* and GEO accession number, are shown together with the ribosomal (*Rib.*), strength (*Str.*) and %GC3 content (*Cont.*) criteria for the scCAI and scnRCA methods, the number of annotated ribosomal proteins (RP#) used for the MILC method, and the genomic %GC content of each species. The ribosomal criterion (*Rib.*) is computed as the mean Z-score of scCAI/scnRCA values for ribosomal proteins against scCAI/scnRCA values for all protein-coding genes in the genome. The strength criterion (*Str.*) is computed as the mean Z-score of scCAI/scnRCA values for the isolated reference set against scCAI/scnRCA values for all protein-coding genes in the genome. The %GC3 content (*Cont.*) criterion is computed as the correlation between scCAI/scnRCA values and gene %GC3 content for all protein-coding genes in the genome (see Carbone *et al.* (2003) *Bioinformatics*,19:16, 2005-2015).

Species	% GC	Method	Average		S#	R#	Rib.	Str.	Cont.	RP#	GEO / Ref.
Bacillus anthracis str Ames Ancestor	43	scCAI	0.36±0.006		4649	3	1.57	2.7	-0.33		GSE22559
		scnRCA	0.40±0.006		4649	3	1.73	2.7	-0.22		GSE22559
		MILC	0.37±0.008		4649	3				57	GSE22559
		CDC	0.23±0.020		4649	3					GSE22559
		δ	0.41±0.004		4649	3				57	GSE22559
Bacillus subtilis 168	43	scCAI	0.24±0.005		3624	4	2.09	3.5	-0.42		GSE37742
		scnRCA	0.30±0.010		3624	4	1.93	3.2	-0.21		GSE37742
		MILC	0.27±0.005		3624	4				63	GSE37742
		CDC	0.05±0.010		3624	4					GSE37742
		δ	0.21±0.008		3624	4				63	GSE37742
Caulobacter crescentus CB15	67	scCAI	0.33±0.008		3641	13	1.01	3.1	0.68		GSE3328
		scnRCA	0.36±0.008		3641	13	2.1	3.9	0.58		GSE3328
		MILC	0.24±0.008		3641	13				55	GSE3328
		CDC	0.15±0.003		3641	13					GSE3328
		δ	0.34±0.010		3641	13				55	GSE3328
Chlamydophila pneumoniae AR39	40	scCAI	0.13±0.004		1077	20	1	3.8	-0.69		GSE7070
		scnRCA	0.25±0.007		1077	20	0.81	3.1	-0.37		GSE7070
		MILC	0.12±0.006		1077	20				53	GSE7070
		CDC	0.04±0.006		1077	20					GSE7070
		δ	0.32±0.007		1077	20				53	GSE7070
Clostridium acetobutylicum ATCC 824	30	scCAI	0.12±0.000		3670	6	1.32	2.5	-0.65		GSE18471
		scnRCA	0.25±0.000		3670	6	1.39	2.7	-0.47		GSE18471
		MILC	0.39±0.006		3670	6				61	GSE18471
		CDC	-0.07±0.007		3670	6					GSE18471
		δ	0.37±0.002		3670	6				61	GSE18471
Clostridium perfringens str 13	29	scCAI	0.35±0.008		2660	6	1.23	2.5	-0.27		GSE12833
		scnRCA	0.42±0.008		2660	6	1.7	2.7	-0.09		GSE12833

		MILC	0.46±0.015		2660	6				61	GSE12833
		CDC	0.05±0.006		2660	6					GSE12833
		δ	0.50±0.005		2660	6				61	GSE12833
Deinococcus radiodurans R1	67	scCAI	0.20±0.006		7845	3	1.05	1.7	0.71		GSE33758
		scnRCA	0.20±0.006		7845	3	1.32	2.1	0.6		GSE33758
		MILC	0.10±0.005		7845	3				56	GSE33758
		CDC	0.11±0.009		7845	3					GSE33758
		δ	0.20±0.005		7845	3				56	GSE33758
Escherichia coli K-12 MG1655	50	scCAI	0.47±0.007		3712	31	1.81	2.8	0.26		[33]
		scnRCA	0.47±0.007		3712	31	1.82	2.8	0.25		[33]
		MILC	0.38±0.007		3712	31				60	[33]
		CDC	0.32±0.005		3712	31					[33]
		δ	0.49±0.007		3712	31				60	[33]
Enterococcus faecalis V583	39	scCAI	0.30±0.000		5974	2	1.53	2.5	-0.4		GSE34432
		scnRCA	0.35±0.000		5974	2	1.62	2.6	-0.33		GSE34432
		MILC	0.41±0.000		5974	2				64	GSE34432
		CDC	0.18±0.018		5974	2					GSE34432
		δ	0.27±0.004		5974	2				64	GSE34432
Haemophilus influenzae Rd KW20	38	scCAI	0.57±0.000		4971	3	1.67	3	-0.4		GSE5061
		scnRCA	0.58±0.000		4971	3	1.76	2.9	-0.27		GSE5061
		MILC	0.43±0.003		4971	3				57	GSE5061
		CDC	0.18±0.004		4971	3					GSE5061
		δ	0.57±0.002		4971	3				57	GSE5061
Lactococcus lactis Il1403	35	scCAI	0.33±0.025		1532	10	1.91	2.9	-0.19		GSE2823
		scnRCA	0.35±0.025		1532	10	2.07	3	-0.12		GSE2823
		MILC	0.27±0.023		1532	10				57	GSE2823
		CDC	0.15±0.015		1532	10					GSE2823
		δ	0.26±0.021		1532	10				57	GSE2823
Listeria monocytogenes EGD-e	37	scCAI	0.37±0.005		5692	4	1.5	2.7	-0.44		GSE22672
		scnRCA	0.38±0.010		5692	4	1.62	2.7	-0.3		GSE22672
		MILC	0.28±0.010		5692	4				58	GSE22672
		CDC	0.19±0.006		5692	4					GSE22672
		δ	0.38±0.005		5692	4				58	GSE22672
Mycobacterium smegmatis MC2-155	67	scCAI	0.42±0.000		841	25*	0.71	1.6	0.9		[33]
		scnRCA	0.44±0.000		841	25*	0.87	1.8	0.8		[33]
		MILC	0.06±0.000		841	25*				58	[33]
		CDC	0.28±0.000		841	25*					[33]
		δ	0.11±0.000		841	25*				58	[33]
Mycoplasma gallisepticum R-low	31	scCAI	0.04±0.004		1413	6	0.2	2.6	-0.93		GSE19755
		scnRCA	0.32±0.016		1413	6	1.26	2.9	-0.3		GSE19755

		MILC	0.17±0.011		1413	6				52	GSE19755
		CDC	0.31±0.014		1413	6					GSE19755
		δ	0.35±0.014		1413	6				52	GSE19755
Myxococcus xanthus DK 1622	69	scCAI	0.32±0.012		21411	3	0.38	1.5	0.91		GSE17912
		scnRCA	0.34±0.012		21411	3	0.56	1.8	0.8		GSE17912
		MILC	0.21±0.012		21411	3				64	GSE17912
		CDC	0.14±0.001		21411	3					GSE17912
		δ	0.37±0.015		21411	3				64	GSE17912
Neisseria gonorrhoeae FA 1090	53	scCAI	0.29±0.017		1727	3	-0.2	1.8	0.7		GSM318543
		scnRCA	0.41±0.012		1727	3	1.28	2.2	0.33		GSM318543
		MILC	0.36±0.008		1727	3				56	GSM318543
		CDC	0.00±0.008		1727	3					GSM318543
		δ	0.38±0.005		1727	3				56	GSM318543
Neisseria meningitidis MC58	51	scCAI	0.12±0.006		3916	3	-0	1.7	0.74		GSE20294
		scnRCA	0.34±0.006		3916	3	1.13	2	0.45		GSE20294
		MILC	0.36±0.019		3916	3				57	GSE20294
		CDC	0.20±0.017		3916	3					GSE20294
		δ	0.43±0.014		3916	3				57	GSE20294
Propionibacterium freudenreichii CIRM-BIA1	67	scCAI	0.18±0.005		9509	4	0.29	1.6	0.83		GSE30841
		scnRCA	0.30±0.010		9509	4	0.75	2.1	0.53		GSE30841
		MILC	0.31±0.009		9509	4				50	GSE30841
		CDC	0.02±0.005		9509	4					GSE30841
		δ	0.33±0.014		9509	4				50	GSE30841
Pseudomonas aeruginosa PAO1	66	scCAI	-0.02±0.000		5543	4	-0.4	1.3	0.8		GSM68692
		scnRCA	0.15±0.000		5543	4	1.14	2	0.47		GSM68692
		MILC	0.16±0.002		5543	4				59	GSM68692
		CDC	0.07±0.001		5543	4					GSM68692
		δ	0.16±0.001		5543	4				59	GSM68692
Pseudomonas fluorescens Pf-5	63	scCAI	-0.05±0.008		14396	6	-0.9	1.4	0.86		GSE16898
		scnRCA	0.22±0.012		14396	6	1.2	2.3	0.32		GSE16898
		MILC	0.23±0.014		14396	6				58	GSE16898
		CDC	0.06±0.009		14396	6					GSE16898
		δ	0.31±0.019		14396	6				58	GSE16898
Pseudomonas putida KT2440	66	scCAI	0.41±0.006		5330	3	0.73	1.6	0.69		GSE24176
		scnRCA	0.44±0.006		5330	3	1.48	2	0.48		GSE24176
		MILC	0.33±0.004		5330	3				58	GSE24176
		CDC	0.22±0.003		5330	3					GSE24176
		δ	0.43±0.005		5330	3				58	GSE24176
Psychrobacter arcticus 273-4	43	scCAI	0.45±0.008		3920	15	1.83	0.3	-0.32		GSE12871
		scnRCA	0.47±0.008		3920	15	1.78	0.3	-0.19		GSE12871

		MILC	0.21±0.018		3920	15				57	GSE12871
		CDC	0.22±0.000		3920	15					GSE12871
		δ	0.38±0.018		3920	15				57	GSE12871
Ralstonia solanacearum GMI1000	67	scCAI	0.13±0.005		2715	4	0	1.2	0.87		GSM832378
		scnRCA	0.29±0.005		2715	4	1.07	1.7	0.67		GSM832378
		MILC	0.20±0.002		2715	4				56	GSM832378
		CDC	0.22±0.002		2715	4					GSM832378
		δ	0.40±0.002		2715	4				56	GSM832378
Rhodococcus jostii RHA1	67	scCAI	0.16±0.000		8042	3	0.33	1.6	0.88		GSE22214
		scnRCA	0.17±0.000		8042	3	0.74	1.9	0.74		GSE22214
		MILC	0.09±0.001		8042	3				59	GSE22214
		CDC	0.02±0.002		8042	3					GSE22214
		δ	0.15±0.001		8042	3				59	GSE22214
Shewanella oneidensis MR-1	45	scCAI	0.27±0.002		8070	20	2.23	3.3	-0.16		GSE3876
		scnRCA	0.29±0.002		8070	20	2.23	3.3	-0.09		GSE3876
		MILC	0.39±0.005		8070	20				62	GSE3876
		CDC	-0.05±0.005		8070	20					GSE3876
		δ	0.26±0.002		8070	20				62	GSE3876
Staphylococcus aureus COL	32	scCAI	0.38±0.007		2719	2	1.72	2.6	-0.45		GSE20973
		scnRCA	0.39±0.007		2719	2	1.83	2.7	-0.37		GSE20973
		MILC	0.40±0.002		2719	2				60	GSE20973
		CDC	0.14±0.007		2719	2					GSE20973
		δ	0.37±0.006		2719	2				60	GSE20973
Streptococcus pneumoniae R6	39	scCAI	0.16±0.006		2017	3	1.94	3	-0.29		GSE21506
		scnRCA	0.23±0.006		2017	3	1.94	3	-0.21		GSE21506
		MILC	0.25±0.004		2017	3				58	GSE21506
		CDC	0.16±0.007		2017	3					GSE21506
		δ	0.22±0.004		2017	3				58	GSE21506
Streptomyces avermitilis MA-4680	72	scCAI	0.14±0.006		7550	3	0.47	1.8	0.86		GSE16892
		scnRCA	0.18±0.012		7550	3	0.8	2.1	0.71		GSE16892
		MILC	0.15±0.002		7550	3				59	GSE16892
		CDC	0.18±0.001		7550	3					GSE16892
		δ	0.15±0.006		7550	3				59	GSE16892
Streptomyces coelicolor A3-2	72	scCAI	0.12±0.006		7810	3	0.52	1.4	0.86		GSE18489
		scnRCA	0.14±0.006		7810	3	1.04	1.9	0.73		GSE18489
		MILC	0.01±0.005		7810	3				62	GSE18489
		CDC	0.14±0.009		7810	3					GSE18489
		δ	0.26±0.007		7810	3				62	GSE18489
Synechocystis PCC 6803	47	scCAI	0.32±0.008		3078	6	1	2.3	0.57		GSE4613
		scnRCA	0.32±0.008		3078	6	0.82	2.1	0.56		GSE4613

		MILC	0.14±0.000		3078	6				56	GSE4613
		CDC	0.09±0.004		3078	6					GSE4613
		δ	0.21±0.004		3078	6				56	GSE4613
Thermotoga maritima MSB8	46	scCAI	0.24±0.007		9150	2	0.77	2.2	0.53		GSE29557
		scnRCA	0.22±0.007		9150	2	0.77	2.2	0.5		GSE29557
		MILC	0.13±0.005		9150	2				54	GSE29557
		CDC	-0.03±0.015		9150	2					GSE29557
		δ	0.24±0.009		9150	2				54	GSE29557
Thermus thermophilus HB8	70	scCAI	0.27±0.006		1907	3	0.41	1.1	0.91		GSE7175
		scnRCA	0.34±0.006		1907	3	0.74	1.6	0.81		GSE7175
		MILC	0.24±0.002		1907	3				57	GSE7175
		CDC	-0.15±0.002		1907	3					GSE7175
		δ	0.49±0.003		1907	3				57	GSE7175
Vibrio cholerae O1 biovar El Tor str. N16961	48	scCAI	0.36±0.008		2717	6	2.25	3.4	-0.17		GSE6468
		scnRCA	0.36±0.004		2717	6	2.2	3.3	-0.13		GSE6468
		MILC	0.30±0.004		2717	6				58	GSE6468
		CDC	0.12±0.009		2717	6					GSE6468
		δ	0.39±0.005		2717	6				58	GSE6468
Yersinia pestis CO92	48	scCAI	0.46±0.004		2955	6	1.93	3	0.03		GSE16898
		scnRCA	0.47±0.004		2955	6	1.91	2.9	0.1		GSE16898
		MILC	0.38±0.004		2955	6				57	GSE16898
		CDC	0.19±0.004		2955	6					GSE16898
		δ	0.42±0.002		2955	6				57	GSE16898
